# Individual variation and plasticity in the infant-directed communication of orang-utan mothers

**DOI:** 10.1098/rspb.2022.0200

**Published:** 2022-05-25

**Authors:** Marlen Fröhlich, Carel P. van Schaik, Maria A. van Noordwijk, Ulrich Knief

**Affiliations:** ^1^ Paleoanthropology, Institute for Archaeological Sciences, Senckenberg Center for Human Evolution and Paleoenvironment, University of Tübingen, Tübingen, Germany; ^2^ Department of Anthropology, University of Zurich, Zurich, Switzerland; ^3^ Comparative Socioecology Research Group, Max Planck Institute of Animal Behavior, Konstanz, Germany; ^4^ Center for the Interdisciplinary Study of Language Evolution (ISLE), University of Zurich, Zurich, Switzerland; ^5^ Department of Evolutionary Biology and Environmental Studies, University of Zurich, Zurich, Switzerland; ^6^ Division of Evolutionary Biology, Faculty of Biology, Ludwig Maximilian University of Munich, Planegg-Martinsried, Germany

**Keywords:** behavioural reaction norm, behavioural flexibility, social context, gestural communication, *Pongo* spp.

## Abstract

Between-individual variation in behavioural expression, such as social responsiveness, has been shown to have important eco-evolutionary consequences. However, most comparative research on non-human primate communication has focused on species- or population-level variation, while among- and within-individual variation has been largely ignored or considered as noise. Here, we apply a behavioural reaction norm framework to repeated observations of mother–offspring interactions in wild and zoo-housed orang-utans (*Pongo abelii, P. pygmaeus*) to tease apart variation on the individual level from population-level and species-level differences. Our results showed that mothers not only differed in the composition of their infant-directed gestural repertoires, but also in communicative tactics, such as gestural redoings (i.e. persistence) and responsiveness to infants' requests. These differences remained after controlling for essential moderators, including species, setting, parity and infant age. Importantly, mothers differed in how they adjusted their behaviour across social contexts, making a strong case for investigating within-individual variation. Our findings highlight that partitioning behavioural variation into its within-individual, between-individual and environmental sources allows us to estimate the extent of plastic responses to the immediate environment in great ape communication.

## 1. Introduction

To better understand the functional role of maternal competence and attachment in human development, researchers commonly draw on studies of mother–offspring relationships in closely related species, in particular non-human primates [1]. Primate mothers’ success in raising offspring may depend on their ability to recognize and respond appropriately to their offspring's signals as well as to guide and coordinate, through the use of signals, their offspring's behaviour [2]. As one of the most effective ways of influencing the behaviour of others [3], communication is the glue that bonds mothers and offspring, which becomes most evident in the coordination of daily routines such as feeding and joint travel.

Individual variation in the way primate mothers respond to their infants has been noted since the earliest detailed records of maternal behaviour [4–6]. Even though sample sizes were typically small for long-lived mammalian species, these field studies reported inter-individual differences in maternal competence, infant handling and maternal rejection, which were attributed partially to maternal experience, but also to mothers' ‘personality’. Later on, more systematic and quantitative studies of mother–infant interactions in both natural and captive settings confirmed that some mothers were consistently more restrictive of their infants' attempts to move out of contact, while others were relatively inattentive and rejecting [7–9].

**Figure 1.**
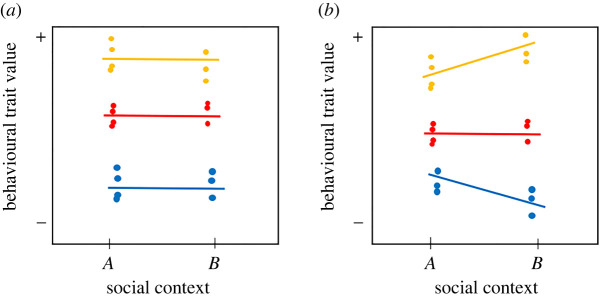
Concepts of behavioural reaction norms. (*a*) Behavioural type: between-individual differences in mean behavioural expression over repeated measures. (*b*) Individual plasticity: individuals differ in their behavioural plasticity across social contexts and there is a positive correlation between an individual's behavioural type (intercept) and its plasticity (slope). Differently coloured lines represent different individuals. Adapted from Hertel *et al*. [18]. (Online version in colour.)

Consistent individual differences (i.e. ‘personality’) in social behaviour, including association patterns or direct physical interactions, have been identified across numerous animal taxa [10] and recently also among great ape species like chimpanzees [11]. Both between- and within-individual variation is increasingly recognized as biologically meaningful [10], which led many behavioural ecologists (mostly working on non-primate species) to shift their focus from population means to the biological underpinnings of variation around means. Consistent individual variation provides the raw material for selection to act upon: without it, there is no opportunity for selection and thus adaptive evolution [12]. Between-individual variation in behavioural expression has been shown to have important eco-evolutionary consequences, for example, to population dynamics, life-history trade-offs and patterns of survival [13], and affects the evolution of behaviours via social evolution [14]. Moreover, accumulating evidence from a variety of species, including humans [15] and other primates [16], indicates that individuals from the same population can differ in behavioural plasticity, which may be due to additive and interactive effects of genetic make-up and past environmental conditions (e.g. early rearing) [17]. A reaction norm framework allows to simultaneously quantify individual variation in average behaviour over repeated observations (personality) *and* individual variation in the degree of behavioural plasticity towards changing environmental conditions [10,18]. Essentially, behavioural variability is partitioned into intrinsic between-individual variation (‘behavioural type’, figure 1*a*) and reversible behavioural plasticity (‘individual plasticity’, figure 1*b*), where the intercept equates to the individual's average behaviour and the slope to its level of plasticity (see also [17]). Thus, a behavioural reaction norm is defined as a set of behavioural phenotypes that a single individual produces in a given set of environmental conditions [10,12].

Primates differ from many other species in that both short- and long-term environmental factors affect the behavioural phenotype. On the one hand, they exhibit great *irreversible* (i.e. developmental) plasticity by producing substantially different behavioural phenotypes depending upon environmental conditions during early life [19]. For example, early long-term deprived ex-laboratory chimpanzees and early maternally deprived zoo chimpanzees showed impaired social grooming activity and thus weaker social integration compared to non-deprived conspecifics [20]. On the other hand, primates are renowned for their remarkable *reversible* plasticity (i.e. behavioural flexibility) allowing individuals to attune to a wide spectrum of immediate social conditions [21]. For example, female vervet monkeys (*Cercopithecus aethiops*) were shown to change their maternal protectiveness when new adult males were introduced to the group [16]. Hence, irrespective of consistent between-individual differences in average levels of behaviour across time and contexts, an individual's social behaviour may independently vary in response to moment-to-moment changes in environmental conditions. This phenomenon can also be studied using the behavioural reaction norm approach introduced above.

Although this approach has been fruitfully applied to social and spatial behavioural patterns of many non-primate species (e.g. [18,22,23]), virtually nothing is known about between-individual variation in both behavioural type and plasticity in the *communicative* behaviour of primates, even though some of the earliest papers on animal personality focused on non-human primates [16,24], and there is evidence for substantial behavioural flexibility in primate communicative behaviour [25]. The scarcity of non-human primate data concerning individual differences in communicative behaviour and other forms of decision-making clearly stems from the logistical and ethical challenges of applying experimental approaches with larger and endangered wildlife. Moreover, primate field studies typically use a small number of individuals, thus essentially ignoring the extent of between-individual differences in signal repertoires and interactional usage (and thus, whether conspicuous communicative patterns are driven by just a few specific individuals). In other words, unexplained variation around the mean is essentially viewed as ‘noise’, which amounts to assuming that individuals would tend towards the same mean value of communicative measures if there are sufficient data. However, movement ecologists recently demonstrated that repeated observations (i.e. GPS fixes) of the same individuals in different environmental settings suffice to investigate variation on the individual level with the variance-partitioning approach introduced above: behavioural variability is partitioned into intrinsic among-individual variation and reversible behavioural plasticity*—*provided the number of individuals and observations is sufficiently large [18].

Partitioning variation in communicative behaviour into its individual and environmental components is critical for at least two reasons: first, to draw correct inferences about the extent of group and possibly even species differences in communicative repertoires and patterns, and second, to assess the role of plastic responses to the immediate (i.e. behavioural flexibility) and developmental environment (ontogenetic plasticity) in great ape communication.

In this study, we aimed at testing the hypothesis of whether mother–offspring communication in orang-utans is characterized by individual variation in behavioural type and plasticity. This approach ensures that the individuals of interest (i.e. mothers) are always interacting with the same social partner (i.e. the current dependent offspring), thus eliminating variation in social relationships. To this end, this study makes use of an existing large-scale dataset collected in wild and captive populations of two different orang-utan species [26,27]. Orang-utans constitute an important model system for such a study, since it has been presumed that orang-utan mothers have a particularly large influence on their infants' behavioural development [28] owing to their rather solitary nature and exceptionally long interbirth intervals [29]. Over the course of infant development, the mother provides the primary model of social and ecological competence, leading to vertical transfer of information critical to survival [30].

Despite their reputation as the ‘solitary great ape’, orang-utans are known to possess a rich repertoire of tactile and visual gestures deployed across a wide range of social contexts (e.g. food sharing, social play and joint travel), both in captive and wild settings (e.g. [27,31]). Experiments have shown that they adopt multimodal tactics to achieve communicative goals based on comprehension, by repeating signals if they are partially understood and switching sensory modalities if completely misunderstood [32], demonstrating a propensity for elaborate and flexible gesture use that parallels that of other great apes. Our previous work on the same large-scale dataset has provided rich evidence for plasticity in communicative behaviour, with signals being flexibly adjusted to both context and partner, and larger repertoires in captivity [26,27]. Specifically, in captivity we found a proliferation of signals whose expression requires flat substrates and involve mobile objects (e.g. somersault, hand-stand, roll on back, hit object and throw object), and in communicative contexts that do not occur on a daily basis in wild settings (e.g. social play and conflict beyond the mother–offspring dyad). Moreover, gestural repertoires of two individuals of the same species living in the same research settings exhibited a larger degree of overlap than those of two individuals living in different settings.

In the light of what we already know about primate mother–offspring relationships, orang-utan mothers will likely adjust their communication according to infant age, their previous mothering experience and the specific behavioural context [4,16,33–35]. For example, some mothers will be much more responsive in the food sharing, but not in the play context, and it would be important to disentangle environmental effects (e.g. captive versus wild setting) from individual ones to understand the extent of between-individual variation in reversible behavioural plasticity. Therefore, the goal of this study is to tease apart effects of individual identity in average behavioural expression as well as individual behavioural change across environmental conditions on one hand, and essential moderators of mother–offspring interactions, such as species, research setting, and infant age on the other. This will allow us to evaluate to which degree communicative behaviour in great apes' communicative exchanges varies between individuals and at the same time is adjusted to varying social conditions.

Specifically, we had two predictions. First, we expected only low to moderate similarity of communicative repertoires (communicative tool-sets) among individual mothers, especially those inhabiting different ecological surroundings (i.e. wild versus captive settings, see above) due to the adaptation to the specific socio-ecological environments individuals interact and grow up in [27]. To address this, we examined the extent to which infant-directed repertoires differed among orang-utan mothers, analysing gestural repertoire similarity between individual mothers living in the same (i.e. both captive or both wild) or different research settings (i.e. captive versus wild). Second, we predicted that orang-utan mothers would significantly differ in specific communicative patterns overall, but also in how these communicative patterns change across social contexts (e.g. food sharing, joint travel and social play). To this end, we adopted a behavioural reaction norm framework by examining individual variation in average behavioural expression (behavioural type) and change across conditions (individual plasticity) in communicative interactions, focusing on two specific measures: gestural redoings (i.e. repeating or elaborating initial gestural signals after communicative failure, frequently referred to as persistence) and responsiveness (i.e. reacting to infants' requests with apparently satisfactory outcomes; *sensu* Hobaiter & Byrne [36]). We here focused on these two aspects since their expression can vary between instances of communicative interactions, as opposed to other parameters like gestural repertoire or modality.

## 2. Material and methods

### (a) Study sites and data collection

This study is based on a pre-existing observational dataset on wild and captive populations of Bornean (*Pongo pygmaeus*) and Sumatran orang-utans (*Pongo abelii*), which was collected between November 2017 and October 2018 at two field sites and five captive facilities (zoos). We observed wild Bornean and Sumatran orang-utans at the long-term research sites of Tuanan (Mawas Reserve, Central Kalimantan, Indonesia) and Suaq Balimbing (Gunung Leuser National Park, South Aceh, Indonesia), respectively. Captive Bornean orang-utans were observed at the zoos of Cologne and Munster, and at Apenheul (Apeldoorn), while Sumatran orang-utans were observed at the zoo of Zurich and at Hellabrunn (Munich). Details on these study sites and data collection have been provided in previous writings [26,27]. In this study, 13 Bornean (9 wild/4 captive) and 13 Sumatran orang-utan mothers were included (8 wild/5 captive; see electronic supplementary material, table S1 for detailed information on subjects and sample sizes per analysis).

This was a purely observational study on wild and zoo animals. Research protocols were approved by the Ministry of Research and Technology (RISTEK; permit no.: 398/SIP/FRP/E5/Dit.KI/XI/2017) and complied with the legal requirements of Indonesia.

### (b) Coding procedure

This study is based on 4839 high-quality video recordings of communicative acts (wild: 3467, captive: 1463) exchanged within mother–offspring pairs which were previously coded using the program BORIS v. 7.0.4. [37]. Specifically, this study mainly focuses on gestural signals, defined as socially directed, mechanically ineffective movements of the extremities, head or body, or body postures (e.g. [38]), thus including both manual and bodily acts. The dataset used for this study also includes facial expressions, but due to their low overall proportion within mother–offspring interactions (*n* = 49, 1% of the dataset) we henceforth refer to all communicative acts as gestures. As detailed in Fröhlich *et al*. [27], *gesture types* were defined and coded based on previous studies on orang-utan communication in captive and wild settings. In addition to signal types, we also coded whether gestures were part of a *sequence* (i.e. redoings *sensu* [39]), which includes instances where initial gesture types were repeated (i.e. simple and exaggerated repetitions) and those where they were replaced by different gesture types in the same or different sensory modality (i.e. elaborations; see electronic supplementary material, table S2 for all original levels of these three coding variables). We also coded the ‘*presumed goal’* as the social context (co-locomote, food share, groom, play/affiliate, move away, sexual contact and stop action [26,27,31]) and the *interaction outcome* (i.e. whether the signaller ceased communication and if it represented the signaller's plausible social goal; [36]). To ensure inter-observer reliability, we evaluated the coding performance of all observers using the Cohen's Kappa coefficient [40]. All trained observers were blind to the study's aims. A detailed overview of individual observers, the study groups coded and final inter-observer reliability scores for our key variables is provided in the electronic supplementary material, table S3.

### (c) Statistical analyses

#### (i) Repertoire overlaps within and between research settings

To assess between-individual variation in gestural repertoire and compare repertoire similarity within and between research settings, we calculated Dice coefficients *D_C_* [41] for each pairing of individuals (see electronic supplementary material, methods; [27,33]). We conducted matrix permutations (*N* = 1000 permutations) in R v. 4.0.3 [42] to assess whether (i) mothers of the same settings (wild-wild and captive-captive pairings) shared more types of signals than mothers living in contrasting settings (wild-captive pairings) and (ii) mothers living in captive settings had more dissimilar repertoires than individuals in wild settings. We predicted this contrast because we included more different captive than wild study groups per species (two or three zoos versus one field site), but also because social life in captivity is more terrestrial (i.e. freeing hands for communication) and more diverse in terms of every-day partner variety (see also [27]). We only included subjects that contributed more than 30 gestural instances to the dataset (i.e. approximate value for which cumulative repertoires approached an asymptote, see Fröhlich *et al*. [26]), and only considered gesture types that were used at least twice by each subject, to obtain more conservative measures of the size and composition of individuals' customarily used repertoires. This method led to the exclusion of seven ‘undersampled’ individuals, leaving 10 Bornean and nine Sumatran mothers for the analysis of repertoire similarity. In the matrix permutation test, we used significance thresholds of *p* ≥ 0.975 and *p* ≤ 0.025, because differences between contrasting groups could either be negative or positive. The distribution of differences between contrasting groups does not necessarily need to be symmetric around zero, which means that we could not adopt the more conventional *p* ≤ 0.05 *p*-value cut-off.

#### (ii) Individual differences and reversible plasticity in communicative interactions

Applying the behavioural reaction norm framework derived from behavioural ecology [10,17], we used repeated observations of individual behaviour to partition variability and thus decompose the phenotypic variance in communication patterns into its between-individual and within-individual sources. We quantified (i) between-individual variation in behavioural types (i.e. differences in average behavioural expression) and (ii) between-individual variation in reversible behavioural plasticity (i.e. differences in how behaviour is shifted between social contexts).

Specifically, we analysed the usage of two different communicative ‘tactics’ of orang-utan mothers, both coded as binary response variables: *redoings* as the production of repeated or modified gestural signals after previous communicative failure [39], and *responsiveness* to infant requests as the occurrence of apparently satisfactory outcomes [36]. We fitted generalized linear mixed models [43] with a binomial error structure and logit link function to examine sources of variation in (i) gestural redoings (*n* = 24 mothers, since two individuals were only recipients, never signallers) and (ii) responsiveness in interactions with their infants (*n* = 26 mothers). In both models, we included the following fixed effects: research setting (two levels: captive and wild), orang-utan species (two levels: Bornean and Sumatran), infant age (in years; covariate with range = 1–7) and parity (number of previous offspring reared at least until juvenility plus present infant; covariate with range = 1–6). We also included social context as a fixed effect, but distinguished only two levels for the sake of simplicity: co-locomote versus other in the redoings model (since this is the major non-play context of infant-directed signalling for mothers), begging versus other in the responsiveness model (since this is the major context of mother-directed signalling for infants; see electronic supplementary material, table S4 for distribution of data across all social contexts). Mother's identity and group identity (i.e. name of zoo or field site) were included as random effects, allowing the mean behavioural expression (i.e. intercept) to vary among individuals.

We first calculated repeatability (i.e. variance standardized individual variation in focal behaviour; R) for our two response variables (gestural redoings and responsiveness) by calculating intraclass correlation coefficients (ICC), which are commonly used in behavioural ecology to assess the repeatability of behavioural traits within individuals [44]. An ICC estimates the amount of variation in the response variable explained by random effects or grouping factors in mixed hierarchical models, and we calculated it using the R package rptR v. 0.9.22 [45], which also implements non-Gaussian models (i.e. Poisson and binomial).

By fitting random intercepts for individual identity and individual random slopes for the ‘environmental gradient’, we then tested if individuals differ in how they shift their communicative tactics across social contexts. Specifically, we compared the Akaike information criterion (AIC) values of two models: one with a random intercept for mother's identity and one with a random intercept for mother's identity *and* a random slope over social context for each individual (i.e. interaction between the social context and mother's identity as a random effect; ‘random regression’ models; see also [18]). Following [18], we used the AIC for model comparison, with a smaller AIC indicating a better predictive model performance. If the difference in AIC between the two models (ΔAIC) is larger than 7, we can infer that the more complicated model indeed provides a better fit [46].

All models were implemented in R v. 4.0.3 [42] using the function *glmer* of the package lme4 [47]. To check for collinearity between predictor variables, we determined the variance inflation factors (VIF; [48]) from a model including only the fixed main effects using the function *vif* of the R package car [49]. This revealed that collinearity was not an issue (max VIF = 1.7). To test whether individual identity played a statistically significant role, we also compared the full models with a reduced model lacking the mother's identity random intercept and environmental gradient random slopes using a likelihood ratio test (LRT) [50].

## 3. Results

### (a) Individual variation in gestural repertoires

**Figure 2.**
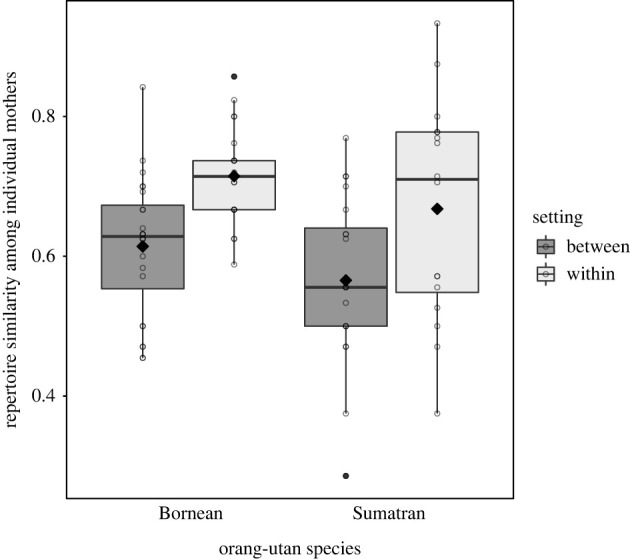
Repertoire similarity between pairs of mothers living in different (between) and the same (within) research settings, separately for each orang-utan species. Indicated are dyadic Dice coefficients (circles), population means (filled diamonds), medians (horizontal lines), quartiles (boxes), percentiles (2.5% and 97.5%, vertical lines) and outliers (filled dots). Individuals may have contributed to multiple data points. (Online version in colour.)

First, we examined to what extent the repertoires of individual orang-utan mothers differed between captivity and the wild. We thus calculated Dice coefficients and conducted matrix permutations tests to analyse whether within-setting repertoire similarity (i.e. similarity of repertoires between two individuals living in the same research setting, that is captivity or wild) differed from between-setting repertoire similarity, separately for Bornean and Sumatran orang-utan mothers. For both species, we found the expected low to moderate overlaps in infant-directed repertoires between individuals, with particularly low similarity between Sumatran mothers living in different settings (Bornean: mean within-D_c_ = 0.71, between-D_c_ = 0.61; Sumatran: within-D_c_ = 0.67, between-D_c_ = 0.57; see figure 2). More importantly, matrix permutation tests showed that the within-setting similarity of communicative repertoires was significantly higher than the between-setting similarity in both species (Borneans: *p* < 0.001, Sumatrans: *p* = 0.006; see figure 2). By contrast, degrees of repertoire overlap within captivity and within the wild did not significantly differ (Bornean: mean within-D_c(wild)_ = 0.7, within-D_c(captive)_ = 0.72, matrix permutation test: *p* = 0.353; Sumatran mothers: within-D_c(wild)_ = 0.74, within-D_c(captive)_ = 0.54; matrix permutation test: *p* = 0.047; see methods regarding lower significance threshold). The latter results should be viewed with caution, however, since sample sizes within specific settings were obviously small (e.g. only four Bornean and four Sumatran mothers contributed to the within-captivity score).

Consistent with a previous study including interactions beyond the mother–offspring dyad [27], our results suggest that communicative repertoires used in the wild and in captivity systematically differ in composition due to enhanced terrestriality and more persistent association with others in zoo settings. However, given that repertoire similarity was overall only low to moderate, this also provides preliminary support for the notion that orang-utan mothers substantially differ in their communicative tool-set deployed in infant-directed communication, regardless of species or setting.

### (b) Individual variation in behavioural type

Next, we investigated whether individual orang-utans differed in communicative patterns on average, by first fitting the model with random intercepts only. LRTs comparing the full model with the respective null model (lacking individual identity as a random effect) revealed that the full model explained behavioural variation significantly better for both response variables (LRT redoings: $x_{1}^{2}=10.236$, *p* = 0.001, *n* = 650; LRT responsiveness: $x_{1}^{2}=15.065$, *p* = 0.001, *n* = 3446). The only significant fixed effect concerned social context: responsiveness to infant requests was significantly higher in begging versus non-begging interactions (see electronic supplementary material, table S5).

**Figure 3.**
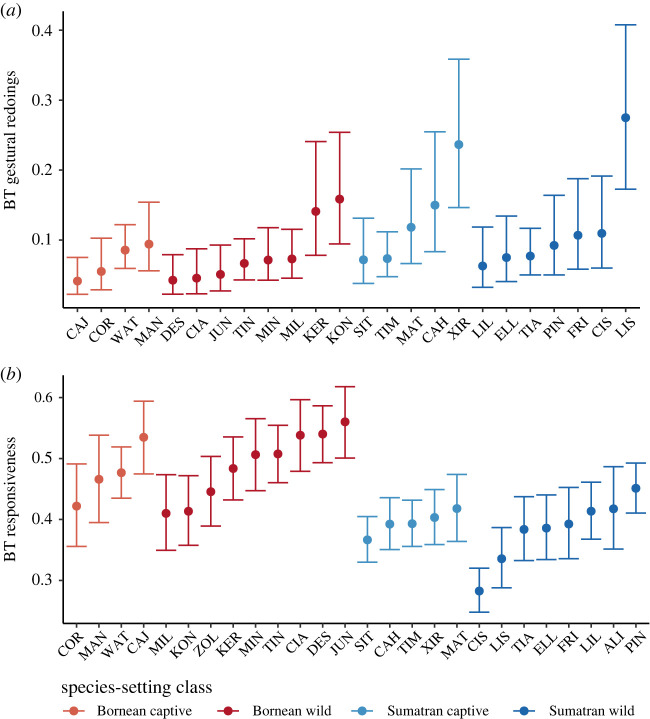
Between-individual variation in behavioural types (BTs) for (*a*) gestural redoings and (*b*) responsiveness in Bornean and Sumatran orang-utan mothers. Plotted are individual random effect coefficients (best linear unbiased predictors, BLUPs) from models examining variation in gestural redoings (*a*) and responsiveness to infant requests (*b*). Colours represent different species-setting combinations. (Online version in colour.)

For gestural redoings, we found significant repeatability on the individual level (*R* = 0.077, s.e. = 0.039, CI = [0, 0.151], *p* < 0.001) but not on the group level (at least not beyond the variance among individuals; *R* = 0.07, s.e. = 0.04, CI = [0, 0.14], *p* = 0.274). For responsiveness, we found significant repeatability on the individual level (*R* = 0.017, s.e. = 0.009, CI = [0, 0.036], *p* < 0.001), as well as on the group level (*R* = 0.114, s.e. = 0.049, CI = [0, 0.173], *p* < 0.001). This means that on average 8% and 2% of the remaining variance (after controlling for confounding effects of species, setting, parity and infant age, see full model output in the electronic supplementary material, table S5) in gestural redoings and responsiveness, respectively, can be attributed to differences between individuals (figure 3). In other words, some orang-utan mothers always persisted more in communicative attempts and were more responsive compared to other mothers (figure 3), and this difference was not caused by differences between orang-utan species or infant developmental stage. While repeatability of the analysed communicative tactics seems fairly low, we need to consider that simple random intercept models do not control for within-individual variation in relation to social context.

### (c) Individual variation in reversible behavioural plasticity

To examine whether orang-utan mothers differ in how they shift communicative behaviour across social conditions, we compared two models for both response variables (i.e. redoings and responsiveness): one with a random intercept for individual identity and one with a random intercept for individual identity *and* a random slope over social contexts for each individual (i.e. interaction between the social context and individual identity as a random effect). A comparison of AIC values revealed that the more complex model including the interaction between social context and individual identity fits better (redoings: ΔAIC = 12.9; responsiveness: ΔAIC = 80).

**Figure 4.**
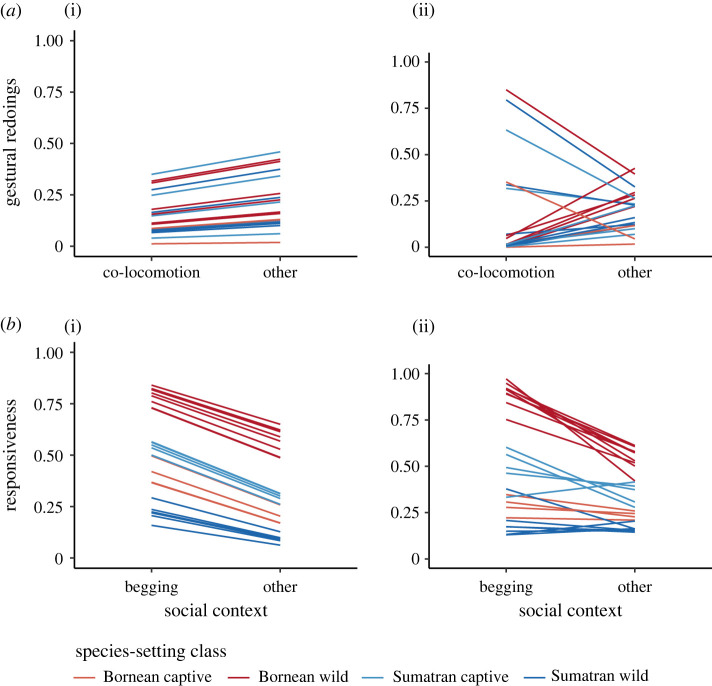
Individual shifts in orang-utan mothers' communicative behaviour and responsiveness across social contexts. (*a*) Gestural redoings in joint travel versus non-travel contexts. (*b*) Maternal responsiveness to infant requests in food begging versus non-begging contexts. (*a*i) and (*b*i) depict prediction lines assuming orang-utan mothers adjust their behaviour equally between social contexts (random intercept). Because predictors were back-transformed, the lines are not strictly parallel as they are on the logit-scale (i.e. only the intercepts vary). (*b*i) and (*b*ii) depict prediction lines assuming orang-utans differ in the extent to which they change behaviour between conditions (random intercept and slope). Colours represent different species-setting combinations. (Online version in colour.)

Our data on individual shifts of communicative behaviour between social contexts thus suggest that not all individuals increase their redoings or responsiveness across relevant social contexts (i.e. co-locomotion or food solicitation) in a similar way. In fact, there are a few individuals that persist more in the co-locomotion context and a few that seem to persist less in this context compared to others (figure 4*a*). While responsiveness to infant requests overall (i.e. on the population and species level) was significantly higher in begging versus non-begging interactions (see electronic supplementary material, table S5), some mothers seemed to drastically reduce responsiveness in non-begging contexts, whereas there was only a slight decrease for others (figure 4*b*). Random regression models account for these differences in reversible behavioural plasticity across social contexts.

## 4. Discussion

In comparative research on primate communication, among- and within-individual behavioural variation has been largely considered as noise. Here, we conducted dissimilarity analyses and applied a behavioural reaction norm framework to communicative interactions in orang-utans, to differentiate between variation reflecting individual differences and variation due to varying external conditions. Analyses of repertoire (dis)similarity revealed that mothers only moderately overlapped in their gestural repertoires, especially when they lived in different settings. Moreover, applying a variance-partitioning approach, we found that mothers differed consistently in two distinct communicative tactics: gestural redoings (i.e. ‘persistence’) and responsiveness to infants' requests. These differences remained even after controlling for essential moderators—species, setting, parity and infant age. Our results thus suggest the existence of consistent individual differences in communicative tactics. Finally, the finding that mothers differed in how they adjusted their behaviour over social contexts makes a strong case for the existence of non-random within-individual variation.

The result that both Bornean and Sumatran orang-utan mothers living in the same research setting have more similar infant-directed repertoires than those of opposing living conditions is not surprising, as it is consistent with previous results on orang-utan repertoires [27]. Captivity constitutes a more sociable and terrestrial living condition for orang-utans of both species, which means that communicative affordances are different from those in the wild. This seems to lead to a number of captivity- and wild-specific signal types as an expression of behavioural plasticity. It is, however, noteworthy that levels of similarity never went above moderate values (i.e. greater than 0.7), suggesting that orang-utan mothers consistently differ from each other in their communicative tool-sets (see also [51]) to coordinate social actions with their infants.

Using a variance-partitioning approach (e.g. [12,18]), differences between mothers became even clearer, because it allowed us to control not only for orang-utan species and research settings, but also infant age, parity and social context. Individual identity also had a significant effect on mothers’ gestural redoings and their responsiveness to infant requests. Recent work on wild chimpanzees has demonstrated long-term repeatability in several social behaviours [11], in line with a large body of work on non-primate species, so one could argue that our findings are to be expected. However, primates are renowned for their exceptional reversible plasticity, flexibly adjusting their behaviour to social circumstances and ecological conditions, presumably to maximize the fitness benefits of social living [52,53]. Hence, consistency in decision-making processes regarding social interactions should not be taken for granted, especially as there is currently little evidence for it [11,15]. Indeed, within-individual variation is not to be underestimated, as exemplified by our findings: the models containing random slopes (i.e. allowing individuals to differ in the slopes of their responses) fitted the data significantly better, providing evidence that mothers differ in how they shift their behaviour across social contexts and thus for significant within-individual variation. What precisely does this mean for our sample of mother–offspring interactions? Responsiveness of mothers to infant requests in general was profoundly larger in begging compared to non-begging contexts, which should be expected given the adaptive benefit of sharing food with kin [54,55]. Importantly, when we allow the effect of individual identity to vary across conditions (i.e. contexts), we find that individuals do not respond in the same way to contextual changes: some mothers show a steeper decrease in responsiveness than others, and for a few individuals, the difference between conditions is almost non-existent. The important conclusion we can derive from these findings is that communicative behaviour and social responsiveness in day-to-day communicative exchanges may vary profoundly between individuals and is *simultaneously* highly flexible.

Since great apes’ communication is strongly tailored to the recipient, behavioural differences between individual infants will of course also contribute to the variation observed within orang-utan mothers (‘social partner effects’ [56]). In other words, variation may be due to mother or infant or the way their interactions happened, and thus how their relationship has developed over time. Thus, it would not be appropriate to attribute such variation to ‘maternal styles’ [57] without further investigation of the mother–offspring relationship, but the important point is that such variation still shows plasticity. We presume that earlier work on maternal styles may have underestimated this interactional component and prematurely generalized toward mothering styles, when the variation was in fact due to how mothers respond to infants of different sex or health status. Moreover, current infant age may not be an ideal proxy for the developmental stage of the mother's offspring, since captive ape infants are probably developing faster and need less locomotory assistance than in the wild. Therefore, the extent to which the differences we found here are due to mothers' personalities or those of their infants remains not entirely clear and can only be solved by collecting data on several consecutive infants; this would be an insightful albeit challenging task, given the slow life history characterizing the great apes, in particular orang-utans [29,58].

Another caveat of this study is that it did not include vocalizations, due to several methodological constraints, discussed in [27], hampering comparability between research settings. Although probably less relevant for maternal communication (great ape mothers only seldom vocalize in response to their infant's vocalizations [2]), a study on stumptail macaques (*Macaca arctoides*) showed that individual differences in maternal responsiveness to infant calls were related to variation in the tendency for infants to leave the mother [59]. Moreover, orang-utan mothers of some populations were shown to use distinct, potentially culturally learned vocalizations (e.g. throat scrape, harmonic uuh) to call their infants [60]. In any case, the possible omission of relevant mother–offspring communication data concerns all individual subjects of this study equally and therefore does not affect our central conclusion: not only individual identity matters for the quantification of communicative output, but also the social context in which individuals are observed.

There are two central concepts in behavioural ecology research on individual differences that have not been analysed in the current study, mainly due to our relatively small sample size: predictability (i.e. residual within-individual variance; [61]) and behavioural syndromes (i.e. a suite of traits correlated at the population or species level; [62]). Both predictability and behavioural syndromes (also referred to as ‘coping styles’) have been shown to have important ecological and evolutionary implications [62]. For primate behaviour, next to nothing is known about the existence of behavioural syndromes. De Lathouwers & Van Elsacker [35] described the maternal styles of protectiveness (associated with contact-making, approaching and restraining the infant), distance (breaking contact and leaving) and refusal (rejecting and nipple-rejecting) in captive bonobos (*Pan paniscus*) and chimpanzees (*Pan troglodytes*). While both species scored similarly on protectiveness, differences were found for distance and refusal behaviours. The authors argued that these interspecies differences in maternal styles mirror species-specific infant development, infant vulnerability to aggression and female sociality. However, this study did not investigate these behavioural correlations on the individual level (i.e. whether between-individual correlations in behaviours exist). Studying whether communicative behaviours are structured into behavioural syndromes will be a fruitful avenue for further research, since it would allow getting better insight into the existence of maternal communication styles in great apes. Such evidence would matter for at least two important reasons. First, comparing correlated suites of communicative behaviours among individuals of different species living in the same setting (i.e. captivity) would be highly insightful for revealing the variation of reversible behavioural plasticity across species. Second, if communicative patterns are organized in behavioural syndromes, they are restricted in their potential to evolve independently [63], which also has implications for the evolution of human communication.

In sum, with this study, we make a case for not only considering between-individual, but also within-individual variation in communicative behaviour. On the one hand, we demonstrated that there is significant between-individual variation in addition to the expected effects of orang-utan species, research setting and other external variables. On the other hand, orang-utan mothers demonstrated reversible plasticity (i.e. behavioural flexibility) by differing in how they shift their behaviour across social contexts. Thus far, studies on primate communication analyse behaviour mainly on the population level, but communicative patterns detected in a population or social community may be driven by just a few specific individuals. Therefore, only by partitioning behavioural variation into its within- and between-individual as well as environmental components can we assess the role of plastic responses to the immediate environment in great ape communication and understand whether such behavioural flexibility has potential to evolve.

## Data Availability

Data and original code have been deposited at Zenodo [64]. Electronic supplementary material is available online [65].
